# Carbapenem-Nonsusceptible Gram-Negative Pathogens in ICU and Non-ICU Settings in US Hospitals in 2017: A Multicenter Study

**DOI:** 10.1093/ofid/ofy241

**Published:** 2018-09-21

**Authors:** Eilish McCann, Arjun Srinivasan, C Andrew DeRyke, Gang Ye, Daryl D DePestel, John Murray, Vikas Gupta

**Affiliations:** 1 Merck & Co., Inc., Kenilworth, New Jersey; 2 Division of Healthcare Quality Promotion, Centers for Disease Control and Prevention, Atlanta, Georgia; 3 Becton, Dickinson and Company, Franklin Lakes, New Jersey

**Keywords:** carbapenems, surveillance, Gram-negative pathogens, susceptibility, intensive care unit

## Abstract

**Background:**

Infections caused by Gram-negative pathogens resistant to carbapenems have limited treatment options and are associated with increased morbidity and mortality. We evaluated the rates, infection sources, and pathogen types associated with carbapenem-nonsusceptible (Carb-NS) Gram-negative isolates in intensive care unit (ICU) and non-ICU settings in a large US hospital database.

**Methods:**

We conducted a retrospective cross-sectional analysis of carbapenem susceptibility of all nonduplicate isolates of Gram-negative pathogens collected from January 1, 2017, to December 31, 2017, at 358 US hospitals in the BD Insights Research Database. Carb-NS isolates included all pathogens reported at the institutional level as intermediate or resistant.

**Results:**

Of 312 075 nonduplicate Gram-negative isolates, 10 698 (3.4%) were Carb-NS. Respiratory samples were the most frequent source of Carb-NS isolates (35.2%); skin/wound accounted for 23.6%. *Pseudomonas aeruginosa* was the most common Carb-NS pathogen (58.5% of isolates), and about 30% were Enterobacteriaceae. The highest rates of Carb-NS were found in *Acinetobacter* spp. (35.6%) and *P. aeruginosa* (14.6%). The rate of Carb-NS was significantly higher in ICU (5.4%) vs non-ICU settings (2.7%; *P* < .0001 in univariate analysis). This difference remained significant in multivariable analysis after adjusting for infection and hospital characteristics (odds ratio, 1.35; 95% confidence interval, 1.17–1.56; *P* < .0001).

**Conclusions:**

Infections caused by Carb-NS isolates pose a significant clinical problem across different sources of infection, species of pathogen, and hospital settings. Widespread infection prevention and antimicrobial stewardship initiatives, in combination with new treatment options, may be required to reduce the burden of carbapenem resistance in health care settings.

First introduced in 1985, carbapenems continue to play a critical role as some of the agents of last resort for the treatment of antibiotic-resistant Gram-negative pathogens [1]. Increasing resistance to carbapanems thus jeopardizes patient outcomes and results in a significant economic burden [2, 3]. The increased morbidity and mortality associated with antibiotic resistance is of particular concern in vulnerable populations such as patients in intensive care units (ICUs) [4]. In recognition of this critical risk to public health, the Centers for Disease Control and Prevention has identified carbapenem-resistant Enterobacteriaceae (CRE) as an urgent threat [5], and the World Health Organization has prioritized the development of antibiotics against CRE and carbapenem-resistant *Acinetobacter baumannii* and *Pseudomonas aeruginosa* [6].

Hospital antibiograms and susceptibility data provide key information about the ecology of Gram-negative pathogens and the prevalence of resistance. This information can inform treatment decisions when selecting appropriate empiric treatments and therapies to address confirmed cases of carbapenem resistance. Effective therapy is critical for successful treatment of patients presenting with serious infections [7]. The objective of this study was to use a large multicenter, real-world database to evaluate carbapenem nonsusceptibility (Carb-NS) in Gram-negative bacteria in both ICU and non-ICU settings in US hospitals, with the goal of gaining insight into common culture sources and settings for infection and key pathogens.

## METHODS

### Study Design

This was a retrospective cross-sectional study of antimicrobial susceptibility of all nonduplicate (first isolate in 30 days) Enterobacteriaceae, *P. aeruginosa*, and *Acinetobacter* spp. isolates from ICU and non-ICU patients collected from January 1, 2017, to December 31, 2017. Reporting institutions comprised 358 US hospitals included in the BD Insights Research Database (Becton, Dickinson and Company, Franklin Lakes, NJ). The electronic surveillance system and clinical research database (formerly the CareFusion Clinical Research Database) have been previously described [8–10]. This database provides good geographical representation across the United States and includes both small and large hospitals in urban and rural areas.

The analyses reported here include all nonduplicate Enterobacteriaceae, *P. aeruginosa*, and *Acinetobacter* spp. (*A. baumannii* and *A. haemolyticus*) isolates from blood, respiratory, urine, skin/wound, intraabdominal, and other sources. Isolates from each source were considered separately; for example, if the patient had a blood and respiratory isolate for *P. aeruginosa* within 30 days, then an isolate was counted for each source. Isolates from the same patient within 30 days were included if they had different drug susceptibilities (>1 susceptibility difference). Isolates were classified as Carb-NS based on facility reports of intermediate susceptibility or resistance to at least 1 of the following agents: (a) ertapenem, imipenem, meropenem, or doripenem for *Escherichia coli*, *Klebsiella pneumoniae*, *Enterobacter aerogenes*, *Enterobacter cloacae*, *Serratia marcescens*, and *Citrobacter freundii*; (b) ertapenem, meropenem, or doripenem for *Proteus mirabilis* and *Morganella morganii*; and (c) imipenem, meropenem, or doripenem for *P. aeruginosa* and *Acinetobacter* spp.

 Care settings were classified using the Centers for Disease Control and Prevention (CDC) National Healthcare Safety Network classification and further classified as ICU (critical care) and non-ICU (inpatient adult wards, specialty care areas, and step-down wards). Hospital-onset isolates were defined as those occurring >3 days after inpatient admission or within 14 days of previous discharge, whereas admission isolates were defined as those occurring ≤3 days of inpatient admission with no previous admission within the past 14 days. Admission period isolates were classified as ICU-associated if the isolate was collected in the admission period and the patient was admitted to an ICU within 3 days of inpatient admission, and they were classified as non-ICU-associated if the isolate was collected in the admission period and the patient was admitted to a non-ICU location within 3 days of inpatient admission and did not have an ICU admission within 3 days of the inpatient admission. Hospital-onset isolates were classified as ICU-associated if the patient was admitted to an ICU on the specimen collection date and as non-ICU-associated if the patient was admitted to a non-ICU location on the specimen collection date with no ICU admission on that date. The study was approved by the New England Institutional Review Board (Wellesley, MA).

### Outcomes

The primary outcome was the rate of Carb-NS isolates as determined by local laboratory breakpoints and practices per routine clinical standard of care.

### Statistical Analysis

Statistical analysis included descriptive analysis, univariate analysis, and multivariable statistical modeling of the data. The univariate analysis was conducted to examine the associations between Carb-NS and ICU status, as well as other potential factors or confounders, including onset period, culture source of isolates collected, pathogen, and hospital characteristics (teaching status, bed size, urban/rural, and geographic location). Chi-square tests (or Fisher’s exact tests when expected frequency <5) were used to assess statistical significance in the univariate (unadjusted) analysis. In the multivariable (adjusted) analysis phase, we used a generalized linear mixed model (GLMM) method with hospital as a random effect to assess the effect of ICU status on Carb-NS rates. Specifically, the Carb-NS rates were modeled using random intercept logistic regression models with hospital as a random effect. Odds ratios (ORs) with 95% confidence intervals (CIs) were calculated. All analyses were conducted using SAS, version 9.4 (SAS Institute, Cary, NC).

## RESULTS

Of the 312 075 nonduplicate isolates tested, 80 310 (25.7%) were collected in the ICU setting and 74 991 (24.0%) were from hospital-onset infections (Table 1). The most common source was urine (59.4%), followed by skin/wound, respiratory, and blood. Over 90% of isolates were collected in urban hospitals. The most common pathogens isolated were *E. coli* (47.9%), *K. pneumoniae* (15.5%), and *P. aeruginosa* (13.7%) (Table 2).

**Table 1.** Distribution of Isolates, Carb-NS Rates, and Univariate Analysis Results

	Total Isolates		Carb-NS Isolates		*P* Value^a^
	No.	%	No.	%	
Overall	312 075	100.0	10 698	3.4	
ICU status					<.0001
Non-ICU	231 765	74.3	6344	2.7	
ICU	80 310	25.7	4354	5.4	
Onset					<.0001
Admission	237 084	76.0	5606	2.4	
Hospital	74 991	24.0	5092	6.8	
Source					<.0001
Urine	185 339	59.4	3311	1.8	
Skin/wound	48 589	15.6	2521	5.2	
Respiratory	32 778	10.5	3775	11.5	
Blood	30 499	9.8	588	1.9	
Other sources	7969	2.6	362	4.5	
Intra-abdominal	6901	2.2	141	2.0	
Teaching hospital					<.0001
Nonteaching	162 287	52.0	4802	3.0	
Teaching	149 788	48.0	5896	3.9	
Bed size					<.0001
>300	180 097	57.7	7283	4.0	
100–300	114 052	36.5	3026	2.7	
<100	17 926	5.7	389	2.2	
Urban/rural					<.0001
Urban	286 398	91.8	10 163	3.5	
Rural	25 677	8.2	535	2.1	
Geographic region					<.0001
South	136 421	43.7	4781	3.5	
Midwest	80 270	25.7	2828	3.5	
Northeast	51 769	16.6	1927	3.7	
West	43 615	14.0	1162	2.7	

Abbreviations: Carb, carbapenem; ICU, intensive care unit; NS, nonsusceptible.

^a^Univariate analysis of clinical and hospital factors correlating with Carb-NS.

**Table 2.** Carb-NS Rates by Pathogen

Pathogen	Total Isolates		Carb-NS Isolates^a^		
	No.	% of Total	No.	% of Total Isolates	% of Carb-NS Isolates
Any	312 075	100.0	10 698	3.4	100
Enterobacteriaceae	265 781	85.2	3227	1.2	30.2
*Escherichia coli*	149 420	47.9	458	0.3	4.3
*Klebsiella pneumoniae*	48 453	15.5	1375	2.8	12.9
*Proteus mirabilis*	26 585	8.5	131	0.5	1.2
*Enterobacter cloacae*	12 971	4.2	696	5.4	6.5
*Klebsiella oxytoca*	7422	2.4	65	0.9	0.6
*Serratia marcescens*	6553	2.1	176	2.7	1.6
*Enterobacter aerogenes*	5087	1.6	218	4.3	2.0
*Morganella morganii*	4866	1.6	21	0.4	0.2
*Citrobacter freundii*	4424	1.4	87	2.0	0.8
*Pseudomonas aeruginosa*	42 880	13.7	6256	14.6	58.5
*Acinetobacter* spp.	3414	1.1	1215	35.6	11.4

Abbreviations: Carb, carbapenem; NS, nonsusceptible.

^a^ *P* < .0001 for correlation of pathogen with Carb-NS.

### Carbapenem Nonsusceptibility in Hospital Isolates

Overall, 10 698 nonduplicate Carb-NS pathogens were isolated, resulting in a Carb-NS rate of 3.4% (Table 1). The majority (80.3%) of Carb-NS pathogens were reported as resistant, and the remainder (19.7%) had intermediate susceptibility (Supplementary Table 1). Respiratory samples were the most frequent source for Carb-NS isolates (n = 3775; 35.3%), followed by urine (30.9%), skin/wound (23.6%), and blood (5.5%). The highest Carb-NS rates were found in respiratory samples (11.5%), followed by skin/wound (5.2%) and other sources (2.0%).

*P. aeruginosa* accounted for over half (58.5%) of all Carb-NS pathogens, followed by Enterobacteriaceae (30.2%, with *K. pneumoniae* constituting 12.9% of all Carb-NS pathogens and 42.6% of Carb-NS Enterobacteriaceae) and *Acinetobacter* spp. (11.4%) (Table 2). The pathogens with the highest rates of Carb-NS were *Acinetobacter* spp. (35.6%) and *P. aeruginosa* (14.6%) (Table 2). The lowest Carb-NS rates were observed in *E. coli* (0.3%) and *M. morganii* (0.4%). Univariate analysis indicated that Carb-NS rates were significantly influenced by pathogen type (*P* < .0001).

The contributions of different pathogens to Carb-NS rates varied depending on the specimen source (Supplementary Table 2). *P. aeruginosa* was the most common Carb-NS pathogen for all specimen sources except blood and intra-abdominal (second to Enterobacteriaceae). The contribution of *Acinetobacter* spp. to Carb-NS isolates was fairly minor (≤5%) for urine and intra-abdominal sources, but this pathogen accounted for approximately 20% and 15% of Carb-NS isolates for skin/wound and blood, respectively.

Carb-NS rates also varied across geographic regions (Supplementary Table 3). The highest rates for Carb-NS Enterobacteriaceae and *Acinetobacter* spp. were in US Department of Health and Human Services (HHS) Region 2 (2.2% and 53.5%, respectively), which includes New York and New Jersey. The highest Carb-NS *P. aeruginosa* rates were observed in HHS Region 6 (17.1%), which includes states in the south-central region (Arkansas, Louisiana, New Mexico, Oklahoma, and Texas).

The majority of Carb-NS isolates were also multidrug-resistant (64.0% to 97.8%) and nonsusceptible to extended-spectrum cephalosporins (54.8% to 86.2%) and fluoroquinolones (57.4% to 97.7%) (Supplementary Table 4). Piperacillin-tazobactam had better activity against Carb-NS *P. aeruginosa* (34.8% nonsusceptible) than against Carb-NS Enterobacteriaceae (71.9% nonsusceptible) or *Acinetobacter* spp. (51.2% nonsusceptible).

### Carbapenem Nonsusceptibility in ICU vs Non-ICU Settings

**Figure 1.**
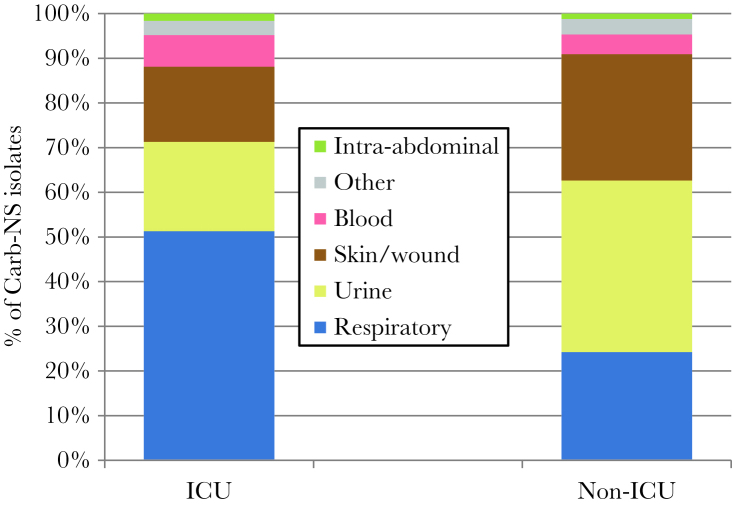
Distribution of Carb-NS ICU and non-ICU isolates by source. Abbreviations: Carb, carbapenem; ICU, intensive-care unit; NS, nonsusceptible.

A total of 4354 Carb-NS isolates (40.7%) were obtained in the ICU, and the remaining 6344 (59.3%) were obtained in non-ICU settings. ICU and non-ICU Carb-NS isolates varied markedly by culture source. The most common source for ICU Carb-NS isolates was respiratory (51.3% vs 24.3% for non-ICU isolates), whereas the most common source for non-ICU Carb-NS isolates was urine (38.5% vs 20.0% for ICU isolates) (Figure 1, Table 3). Skin/wound was a more common source of Carb-NS isolates in non-ICU settings compared with ICUs (28.2% vs 16.8%). In contrast to the differences observed in the source of Carb-NS isolates, pathogen distribution between ICU and non-ICU settings was quite similar. Enterobacteriaceae accounted for 29.0% of Carb-NS isolates in the ICU compared with 31.0% in non-ICU settings. *P. aeruginosa* (58.4% in the ICU vs 58.5% for non-ICU) and *Acinetobacter* spp. (12.6% vs 10.5%) were also fairly equally distributed between the 2 settings (Table 3).

**Table 3.** Adjusted Effect of ICU Status on Carb-NS Rates: Overall and by Onset, Source, and Pathogen

Characteristic or Pathogen	ICU			Non-ICU			ICU vs Non-ICU: Model-Estimated OR^a^			
	NS	Tested	NS%	NS	Tested	NS%	OR	95% CI		*P* Value
Overall	4354	80 310	5.4	6344	231 765	2.7	1.35	1.17	1.56	<.0001
Onset										
Admission	2012	54 795	3.7	3594	182 289	2.0	1.53	1.32	1.78	<.0001
Hospital	2342	25 515	9.2	2750	49 476	5.6	1.19	1.03	1.39	.0223
Source										
Blood	311	11 351	2.7	277	19 148	1.4	1.34	1.09	1.65	.0062
Intra-abdominal	68	2076	3.3	73	4825	1.5	1.54	1.06	2.23	.0236
Other sources	138	2106	6.6	224	5863	3.8	1.66	1.27	2.17	.0002
Respiratory	2234	20 316	11.0	1541	12 462	12.4	0.93	0.80	1.08	.3423
Skin/wound	732	9041	8.1	1789	39 548	4.5	1.63	1.40	1.91	<.0001
Urine	871	35 420	2.5	2440	149 919	1.6	1.18	1.02	1.36	.0292
Pathogen										
Enterobacteriaceae	1263	65 684	1.9	1964	200 097	1.0	1.59	1.44	1.75	<.0001
*E. coli*	153	32 685	0.5	305	116 735	0.3	1.70	1.38	2.09	<.0001
*K. pneumoniae*	612	14 084	4.3	763	34 369	2.2	1.82	1.60	2.08	<.0001
*P. mirabilis*	36	6749	0.5	95	19 836	0.5	1.03	0.70	1.53	.8747
*E. cloacae*	241	3716	6.5	455	9255	4.9	1.23	1.03	1.47	.0237
*K. oxytoca*	24	2046	1.2	41	5376	0.8	1.35	0.81	2.27	.2526
*S. marcescens*	79	2477	3.2	97	4076	2.4	1.36	0.99	1.86	.0570
*E. aerogenes*	84	1756	4.8	134	3331	4.0	1.18	0.88	1.59	.2671
*M. morganii*	5	1161	0.4	16	3705	0.4	0.94	0.34	2.57	.8995
*C. freundii*	29	1010	2.9	58	3414	1.7	1.61	1.02	2.56	.0427
*P. aeruginosa*	2544	13 250	19.2	3712	29 630	12.5	1.55	1.41	1.70	<.0001
*Acinetobacter* spp.	547	1376	39.8	668	2038	32.8	1.38	1.15	1.66	.0006

Abbreviations: Carb, carbapenem; CI, confidence interval; ICU, intensive care unit; NS, nonsusceptible; OR, odds ratio.

^a^Effect of ICU status adjusted using generalized linear mixed models. Adjusting variables include onset, source, pathogen, and hospital characteristics (teaching status, bed size, urban/rural, geographic region).

**Figure 2.**
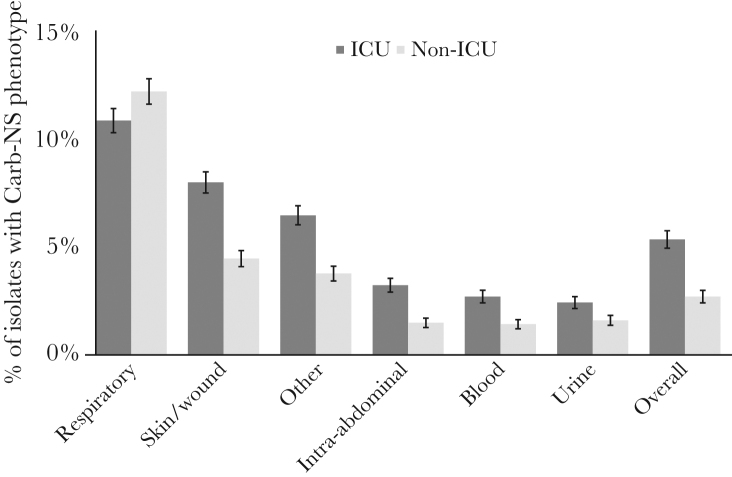
Carb-NS rates by ICU status and isolate source. Capped error bars indicate 95% confidence intervals. Abbreviations: Carb, carbapenem; ICU, intensive care unit; NS, nonsusceptible.

The rate of Carb-NS was significantly higher in ICU settings (5.4%) than in non-ICU settings (2.7%; *P* < .0001 in unadjusted [univariate] analysis) (Table 1). For both ICU and non-ICU settings, the sources with the highest Carb-NS rates were respiratory and skin/wound (Figure 2), and the pathogens with the highest Carb-NS rates were *Acinetobacter* spp. and *P. aeruginosa* (Table 3). In addition to ICU setting, other clinical and hospital factors associated with significantly higher Carb-NS rates included hospital-onset infections, isolate source, teaching vs nonteaching hospital, greater bed size, urban status, and non-Western US geographic region (all *P* < .0001) (Table 1).

### Multivariable Regression Analysis of the Association Between ICU Status and Carb-NS

The 2-fold difference in the Carb-NS rate in ICU vs non-ICU settings could potentially be explained by other factors associated with Carb-NS in univariate analyses. We therefore conducted a multivariable analysis using GLMM to evaluate the impact of the ICU setting on Carb-NS rates in greater detail.

The GLMM analysis, which was adjusted for infection onset, isolate source, pathogen, and hospital characteristics, confirmed that the Carb-NS rate was significantly higher in ICU vs non-ICU settings (OR, 1.35; 95% CI, 1.17–1.56; *P* < .0001). With the exception of respiratory isolates, isolates from all sources had significantly higher Carb-NS rates in ICU vs non-ICU settings (Table 3). The Carb-NS rate was also significantly higher for ICU vs non-ICU settings for both admission-onset and hospital-onset isolates (Table 3).

ICU status had varying effects on Carb-NS for different pathogens. The 3 most common pathogens, *E. coli*, *K. pneumoniae*, and *P. aeruginosa*, all had significantly higher Carb-NS rates in ICU vs non-ICU settings (ORs, 1.70, 1.82, and 1.55, respectively; all *P* < .0001), as did the Enterobacteriaceae family (OR, 1.59) (Table 3; Supplementary Figure 1). Carb-NS rates were also significantly higher in the ICU for *E*. *cloacae*, C*. freundii,* and *Acinetobacter* spp. ICU status did not have a significant effect on Carb-NS rates for the remaining pathogens.

## DISCUSSION

Carbapenems are often used as one of the agents of last resort in the treatment of serious Gram-negative infections; therefore, carbapenem resistance is a critical clinical problem [11]. In the study reported here, we found an overall rate of 3.4% Carb-NS in 312 075 hospital Gram-negative bacterial isolates, with *P. aeruginosa* being the most frequent Carb-NS pathogen. Respiratory samples were the most common source of Carb-NS pathogens; skin/wound isolates accounted for almost one-quarter of isolates. Carb-NS rates were significantly higher in ICU vs non-ICU settings, although greater numbers of Carb-NS were identified in non-ICU settings.

Although almost unheard of in the United States until the 1990s, carbapenem resistance has become an important public health problem [12]. CRE is of particular concern. Not only are Enterobacteriaceae a frequent cause of common infections, but colonization with these bacteria allows efficient transfer of CRE between patients, particularly within health care settings [12]. CRE frequently carry plasmid-mediated carbapenem resistance determinants, such as *K. pneumoniae* carbapenemase (KPC), which can be easily disseminated to different pathogens [13]. The problem of carbapenem resistance in this pathogen is magnified by the paucity of options for the treatment of CRE [11], which may lead to inappropriate initial therapy [14]. At current rates, CRE infections are estimated to result in a 26% mortality rate and cost hospitals $275 million annually [2].

Approximately 30% of the Carb-NS isolates identified in our study were Enterobacteriaceae; the most common individual Enterobacteriaceae pathogens were *K. pneumoniae* and *E. cloacae.* Both pathogens have seen rapid increases in Carb-NS rates in the past few years [15, 16]. In the United States, rates of carbapenem-resistant *K. pneumoniae* increased from 0.1% in 2002 to 4.5% in 2010 [15]. Although less common, *E. cloacae* had the highest Carb-NS rate among Enterobacteriaceae in our analysis. Recent studies have suggested that subsequent to the rapid increase that occurred in carbapenem-resistant *K. pneumoniae*, a “second epidemic” of carbapenem-resistant *E. cloacae* may be occurring [16].

Although early clinical attention was primarily focused on CRE, carbapenem-resistant nonfermenters, particularly *P. aeruginosa* and *Acinetobacter* spp., are now recognized as an increasing problem. These pathogens have always been difficult to treat, but carbapenem resistance further compounds the morbidity and mortality of associated infections [17]. Of note, carbapenem resistance in *A. baumannii* more than doubled between 2003/2005 and 2009/2012 (21.0% to 47.9%) [18]. The high rates of resistance in nonfermenters are of significant concern, as carbapenem resistance is associated with a more than 2-fold increase in mortality for both *P. aeruginosa* (adjusted OR, 2.38) [19] and *Acinetobacter* (adjusted OR, 2.49) [20].

The majority of Carb-NS pathogens in this study were multidrug-resistant and resistant to extended-spectrum cephalosporins, fluoroquinolones, and piperacillin-tazobactam. This finding provides strong support for further investigations into new antimicrobial options with activity against Carb-NS isolates.

Respiratory infections were the most common source of Carb-NS isolates, and *P. aeruginosa* accounted for >70% of the Carb-NS respiratory isolates. Pneumonia is a leading cause of death among hospital patients in the United States [21] and is a particular problem in the ICU. The mean hospital stay for *P. aeruginosa* pneumonia in US ICUs is 55.4 days per patient, and the mortality rate is >20% [22], a frequency that likely increases with carbapenem resistance [19]. These grim statistics highlight the clinical relevance of high Carb-NS rates in respiratory *P. aeruginosa* isolates and the vital need for more therapeutic options to treat carbapenem-resistant Gram-negative respiratory infections.

Our study also highlights skin and wound infections as an important potential source of Carb-NS pathogens. Recognition of Carb-NS pathogens in skin/wound samples is imperative from both the patient management and infection prevention perspectives, as wounds can serve as mobile reservoirs for CRE and other pathogens, thereby increasing their spread within and outside of hospitals [23]. Skin/wound infections are not typically included in routine surveillance of sterile site cultures, and there is thus limited data on carbapenem resistance from this source. In our study, skin/wound isolates showed a high rate of Carb-NS (5.2%; second only to respiratory isolates) and accounted for 23.6% of the total Carb-NS isolates. We hope this finding encourages clinical centers to include skin and wound infections in carbapenem resistance surveillance initiatives.

In our study, ICUs had significantly higher rates of Carb-NS Gram-negative pathogens than non-ICU settings. A significant difference in Carb-NS rates for ICU vs non-ICU settings was observed for all sources except respiratory and for the most common pathogens. It is unclear why respiratory specimens, the most common source of Carb-NS isolates overall and in the ICU and the source with the highest Carb-NS rate, did not show a difference for ICU vs non-ICU settings.

Higher Carb-NS rates for ICU vs non-ICU have also been noted in other studies [24–26]. This is perhaps not surprising given that many of the risk factors for carbapenem-resistant infections, such as antibiotic exposure, underlying diseases, invasive procedures, medical devices, and mechanical ventilation [27], are more common in the ICU than in general wards. The high Carb-NS rates in the ICU may be related to intestinal carriage of carbapenem-resistant Gram-negative bacteria in ICU patients, which have been found to increase rapidly during ICU stays (from 5.6% after 1 week to 58.6% after 6 weeks in the ICU) [28].

The impact of antimicrobial resistance in the ICU is magnified by the critical illnesses faced by this patient population. ICU patients are highly vulnerable to infections, which can be difficult to treat in ICU patients due to comorbidities and altered pharmacokinetic/pharmacodynamic parameters as a result of sepsis or augmented renal clearance [9, 29]. ICU patients are thus at high risk for poor outcomes subsequent to infections with Carb-NS pathogens. Antimicrobial resistance in Gram-negative pathogens has been shown to increase mortality, length of stay, and economic costs in ICU patients [30].

Although Carb-NS rates were higher in the ICU, it is important to note that non-ICU settings had greater overall numbers. These data serve as a reminder that although the ICU may serve as a locus for Carb-NS pathogens, infections are also common in the non-ICU setting. Accordingly, hospital-wide infection control practices are critical for preventing the spread of resistant pathogens. Hospital-onset rates of Carb-NS pathogens were about 2.5-fold higher than the rates of Carb-NS at admission (6.8% vs 2.4%), supporting intensive national efforts to reduce hospital-acquired infections.

Study limitations include the lack of standardization with respect to the use of methods for determining Carb-NS and the lack of a central laboratory to confirm results, as susceptibility results were based on local laboratory practices. As observed recently [13], methods for carbapenem testing vary widely across different clinical centers, which may have influenced the Carb-NS results reported. It is also important to note that data were collected and analyzed from the perspective of unique nonduplicated collected cultures and not from the perspective of unique patients. We were therefore unable to perform adjusted analyses for ICU vs non-ICU based on patient characteristics. Finally, a key limitation of all retrospective studies of antimicrobial resistance, including the one reported here, is that culturing of hospitalized patients relies on the clinician’s assessment of the need for a clinical culture. Accordingly, our findings are based on a potentially biased sample representing more severely ill patients who required clinical culture. We therefore cannot infer the rates of Carb-NS isolates to the hospitalized patient population as a whole, as not all patients provided samples for cultures.

We conclude from our findings that Carb-NS Gram-negative bacteria continue to be an important problem in hospitals. Carb-NS bacteria, especially CRE, are an important focus of national infection control efforts. The CDC has identified CRE as an “urgent” threat to human health [5] and has developed resources to help facilities control the spread of these organisms (https://www.cdc.gov/hai/organisms/cre/cre-toolkit/index.html). The challenge of carbapenem resistance is compounded by the continued emergence and spread of novel carbapenemases [13]. An early and aggressive response to imported novel carbapenemases could potentially prevent them from becoming endemic in the United States. To that end, the CDC has launched a containment strategy that comprises improved laboratory detection of novel carbapenemases and resources to perform colonization testing through the Antimicrobial Resistance Lab Network (AR Lab Network), along with improved response capacity at state health departments (https://www.cdc.gov/hai/containment/guidelines.html). Infectious disease clinicians are critical partners in this control effort as they are the primary point of contact for many patients with Carb-NS infections. They should be aware of AR Lab Network resources and should know which Carb-NS isolates to refer to their state laboratory for further testing.

Although overall Carb-NS rates are fairly low in US hospitals, high rates are observed for some sources such as respiratory, some pathogens such as *P. aeruginosa* and *Acinetobacter* spp., and some locations such as the ICU. Unexpected sources also contribute to Carb-NS isolates. Given the important contributions of skin/wound infections to Carb-NS hospital isolates, it is clear that infection prevention and antimicrobial stewardship initiatives need to address carbapenem resistance associated with all types of infection in both ICU and non-ICU settings. We hope our data will encourage hospitals to conduct evaluations at the facility level that can be used to guide local antimicrobial therapy. In addition to these efforts, continued research into therapeutic options to treat carbapenem-resistant infections is urgently needed, particularly for respiratory infections. A multifaceted approach to this clinically significant problem, as exemplified by the tools developed by the CDC, may help stem the tide of carbapenem resistance.

## Supplementary Data

Supplementary materials are available at *Open Forum Infectious Diseases* online. Consisting of data provided by the authors to benefit the reader, the posted materials are not copyedited and are the sole responsibility of the authors, so questions or comments should be addressed to the corresponding author.

ofy241_suppl_supplementary_material
