# Supplementary material for: Carbapenem-Nonsusceptible Gram-Negative Pathogens in ICU and Non-ICU Settings in US Hospitals in 2017: A Multicenter Study
Source: Open Forum Infect Dis. 2018 Sep 21;5(10):ofy241. doi: 10.1093/ofid/ofy241 (PMC6194421; doi:10.1093/ofid/ofy241)
Supplement: ofy241_suppl_supplementary_material [file ofy241_suppl_supplementary_material.docx]

**Supplementary Information**

**Carbapenem Non-susceptible Gram-negative Pathogens in ICU and Non-ICU Settings in US Hospitals in 2017: A Multicenter Study**

**Supplementary Table S1.** Resistant (Carb-R) and Intermediate Susceptibility (Carb-I) Status by Pathogen.

| **Pathogen** | **Number of Total Isolates** | **Number of Carb-NS Isolates** | **Carb-R** | |  | **Carb-I** | |
| --- | --- | --- | --- | --- | --- | --- | --- |
|  |  |  | **Number of Isolates** | **% of Carb-NS Isolates** |  | **Number of Isolates** | **% of Carb-NS Isolates** |
| Any | 312075 | 10698 | 8593 | 80.3 |  | 2105 | 19.7 |
| Enterobacteriaceae | 265781 | 3227 | 2507 | 77.7 |  | 720 | 22.3 |
| *Escherichia coli* | 149420 | 458 | 352 | 76.9 |  | 106 | 23.1 |
| *Klebsiella pneumoniae* | 48453 | 1375 | 1275 | 92.7 |  | 100 | 7.3 |
| *Proteus mirabilis* | 26585 | 131 | 118 | 90.1 |  | 13 | 9.9 |
| *Enterobacter cloacae* | 12971 | 696 | 447 | 64.2 |  | 249 | 35.8 |
| *Klebsiella oxytoca* | 7422 | 65 | 53 | 81.5 |  | 12 | 18.5 |
| *Serratia marcescens* | 6553 | 176 | 94 | 53.4 |  | 82 | 46.6 |
| *Enterobacter aerogenes* | 5087 | 218 | 91 | 41.7 |  | 127 | 58.3 |
| *Morganella morganii* | 4866 | 21 | 16 | 76.2 |  | 5 | 23.8 |
| *Citrobacter freundii* | 4424 | 87 | 61 | 70.1 |  | 26 | 29.9 |
| *Pseudomonas aeruginosa* | 42880 | 6256 | 4952 | 79.2 |  | 1304 | 20.8 |
| *Acinetobacter* spp. | 3414 | 1215 | 1134 | 93.3 |  | 81 | 6.7 |

Abbreviations: Carb, carbapenem; NS, non-susceptible

**Supplementary Table S2.** Distribution of Pathogens and Carb-NS Pathogens by Specimen Source

| **Specimen source** | **Pathogen** | **Total Isolates** | | |  | | **Carb-NS Isolates** | | | | | |
| --- | --- | --- | --- | --- | --- | --- | --- | --- | --- | --- | --- | --- |
|  |  | **n** | | **% of total isolates for specimen source** |  | | **n** | | **% of total isolates for specimen source** | | **% of Carb-NS isolates for specimen source** | |
| All | All | 312075 | | 100% |  | | 10698 | | 3.4% | | 100% | |
| Urine | Any | 185339 | | 100% |  | | 3311 | | 1.8% | | 100% | |
|  | Enterobacteriaceae | 170681 | | 92.1% |  | | 1442 | | 0.8% | | 43.6% | |
|  | *E. coli* | 108885 | | 58.7% |  | | 265 | | 0.2% | | 8.0% | |
|  | *K. pneumoniae* | 30141 | | 16.3% |  | | 611 | | 2.0% | | 18.5% | |
|  | *P. mirabilis* | 15112 | | 8.2% |  | | 68 | | 0.4% | | 2.1% | |
|  | *E. cloacae* | 4474 | | 2.4% |  | | 311 | | 7.0% | | 9.4% | |
|  | *K. oxytoca* | 3496 | | 1.9% |  | | 24 | | 0.7% | | 0.7% | |
|  | *S.marcescens* | 1342 | | 0.7% |  | | 28 | | 2.1% | | 0.8% | |
|  | *E. aerogenes* | 2454 | | 1.3% |  | | 90 | | 3.7% | | 2.7% | |
|  | *M. morganii* | 2141 | | 1.2% |  | | 5 | | 0.2% | | 0.2% | |
|  | *C. freundii* | 2636 | | 1.4% |  | | 40 | | 1.5% | | 1.2% | |
|  | *P. aeruginosa* | 14155 | | 7.6% |  | | 1731 | | 12.2% | | 52.3% | |
|  | *Acinetobacter* spp. | 503 | | 0.3% |  | | 138 | | 27.4% | | 4.2% | |
| Skin/wound | Any | 48589 | | 100.0% |  | | 2521 | | 5.2% | | 100.0% | |
|  | Enterobacteriaceae | 35899 | | 73.9% |  | | 691 | | 1.9% | | 27.4% | |
|  | *E. coli* | 12069 | | 24.8% |  | | 85 | | 0.7% | | 3.4% | |
|  | *K. pneumoniae* | 5148 | | 10.6% |  | | 290 | | 5.6% | | 11.5% | |
|  | *P. mirabilis* | 7081 | | 14.6% |  | | 44 | | 0.6% | | 1.7% | |
|  | *E. cloacae* | 4288 | | 8.8% |  | | 157 | | 3.7% | | 6.2% | |
|  | *K. oxytoca* | 1749 | | 3.6% |  | | 14 | | 0.8% | | 0.6% | |
|  | *S.marcescens* | 1932 | | 4.0% |  | | 31 | | 1.6% | | 1.2% | |
|  | *E. aerogenes* | 775 | | 1.6% |  | | 37 | | 4.8% | | 1.5% | |
|  | *M. morganii* | 1889 | | 3.9% |  | | 12 | | 0.6% | | 0.5% | |
|  | *C. freundii* | 968 | | 2.0% |  | | 21 | | 2.2% | | 0.8% | |
|  | *P. aeruginosa* | 11403 | | 23.5% |  | | 1338 | | 11.7% | | 53.1% | |
|  | *Acinetobacter* spp. | 1287 | | 2.6% |  | | 492 | | 38.2% | | 19.5% | |
| Respiratory | Any | 32778 | | 100% |  | | 3775 | | 11.5% | | 100% | |
|  | Enterobacteriaceae | 18927 | | 57.7% |  | | 626 | | 3.3% | | 16.6% | |
|  | *E. coli* | 4640 | | 14.2% |  | | 35 | | 0.8% | | 0.9% | |
|  | *K. pneumoniae* | 5669 | | 17.3% |  | | 278 | | 4.9% | | 7.4% | |
|  | *P. mirabilis* | 1547 | | 4.7% |  | | 8 | | 0.5% | | 0.2% | |
|  | *E. cloacae* | 2194 | | 6.7% |  | | 132 | | 6.0% | | 3.5% | |
|  | *K. oxytoca* | 1036 | | 3.2% |  | | 17 | | 1.6% | | 0.5% | |
|  | *S.marcescens* | 2176 | | 6.6% |  | | 83 | | 3.8% | | 2.2% | |
|  | *E. aerogenes* | 1160 | | 3.5% |  | | 53 | | 4.6% | | 1.4% | |
|  | *M. morganii* | 231 | | 0.7% |  | | 3 | | 1.3% | | 0.1% | |
|  | *C. freundii* | 274 | | 0.8% |  | | 17 | | 6.2% | | 0.5% | |
|  | *P. aeruginosa* | 12706 | | 38.8% |  | | 2687 | | 21.1% | | 71.2% | |
|  | *Acinetobacter* spp. | 1145 | | 3.5% |  | | 462 | | 40.3% | | 12.2% | |
| Blood | Any | 30499 | | 100.0% |  | | 588 | | 1.9% | | 100.0% | |
|  | Enterobacteriaceae | 27725 | | 90.9% |  | | 276 | | 1.0% | | 46.9% | |
|  | *E. coli* | 16943 | | 55.6% |  | | 39 | | 0.2% | | 6.6% | |
|  | *K. pneumoniae* | 5136 | | 16.8% |  | | 134 | | 2.6% | | 22.8% | |
|  | *P. mirabilis* | 1996 | | 6.5% |  | | 5 | | 0.3% | | 0.9% | |
|  | *E. cloacae* | 1183 | | 3.9% |  | | 45 | | 3.8% | | 7.7% | |
|  | *K. oxytoca* | 644 | | 2.1% |  | | 4 | | 0.6% | | 0.7% | |
|  | *S.marcescens* | 852 | | 2.8% |  | | 24 | | 2.8% | | 4.1% | |
|  | *E. aerogenes* | 406 | | 1.3% |  | | 19 | | 4.7% | | 3.2% | |
|  | *M. morganii* | 349 | | 1.1% |  | | 1 | | 0.3% | | 0.2% | |
|  | *C. freundii* | 216 | | 0.7% |  | | 5 | | 2.3% | | 0.9% | |
|  | *P. aeruginosa* | 2410 | | 7.9% |  | | 225 | | 9.3% | | 38.3% | |
|  | *Acinetobacter* spp. | 364 | | 1.2% |  | | 87 | | 23.9% | | 14.8% | |
| Other sources | Any | 7969 | | 100.0% |  | | 362 | | 4.5% | | 100.0% | |
|  | Enterobacteriaceae | 6290 | | 78.9% |  | | 117 | | 1.9% | | 32.3% | |
|  | *E. coli* | 3295 | | 41.3% |  | | 20 | | 0.6% | | 5.5% | |
|  | *K. pneumoniae* | 1038 | | 13.0% |  | | 43 | | 4.1% | | 11.9% | |
|  | *P. mirabilis* | 671 | | 8.4% |  | | 4 | | 0.6% | | 1.1% | |
|  | *E. cloacae* | 408 | | 5.1% |  | | 26 | | 6.4% | | 7.2% | |
|  | *K. oxytoca* | 199 | | 2.5% |  | | 4 | | 2.0% | | 1.1% | |
|  | *S.marcescens* | 175 | | 2.2% |  | | 6 | | 3.4% | | 1.7% | |
|  | *E. aerogenes* | 167 | | 2.1% |  | | 12 | | 7.2% | | 3.3% | |
|  | *M. morganii* | 181 | | 2.3% |  | | 0 | | 0.0% | | 0.0% | |
|  | *C. freundii* | 156 | | 2.0% |  | | 2 | | 1.3% | | 0.6% | |
|  | *P. aeruginosa* | 1596 | | 20.0% |  | | 216 | | 13.5% | | 59.7% | |
|  | *Acinetobacter* spp. | 83 | | 1.0% |  | | 29 | | 34.9% | | 8.0% | |
| Intra-abdominal | Any | 6901 | 100.0% | | |  | | 141 | | 2.0% | | 100.0% |
|  | Enterobacteriaceae | 6259 | 90.7% | | |  | | 75 | | 1.2% | | 53.2% |
|  | *E. coli* | 3588 | 52.0% | | |  | | 14 | | 0.4% | | 9.9% |
|  | *K. pneumoniae* | 1321 | 19.1% | | |  | | 19 | | 1.4% | | 13.5% |
|  | *P. mirabilis* | 178 | 2.6% | | |  | | 2 | | 1.1% | | 1.4% |
|  | *E. cloacae* | 424 | 6.1% | | |  | | 25 | | 5.9% | | 17.7% |
|  | *K. oxytoca* | 298 | 4.3% | | |  | | 2 | | 0.7% | | 1.4% |
|  | *S.marcescens* | 76 | 1.1% | | |  | | 4 | | 5.3% | | 2.8% |
|  | *E. aerogenes* | 125 | 1.8% | | |  | | 7 | | 5.6% | | 5.0% |
|  | *M. morganii* | 75 | 1.1% | | |  | | 0 | | 0.0% | | 0.0% |
|  | *C. freundii* | 174 | 2.5% | | |  | | 2 | | 1.1% | | 1.4% |
|  | *P. aeruginosa* | 610 | 8.8% | | |  | | 59 | | 9.7% | | 41.8% |
|  | *Acinetobacter* spp. | 32 | 0.5% | | |  | | 7 | | 21.9% | | 5.0% |

Abbreviations: Carb, carbapenem; NS, non-susceptible

**Supplementary Table S3**. Distribution of Carb-NS Isolates by US Department of Health & Human Services (HHS) Region.

| **HHS region** | **States^a^** | **Carb-NS Enterobacteriaceae** | |  | **Carb-NS**  ***P. aeruginosa*** | |  | **Carb-NS**  ***Acinetobacter* spp** | |
| --- | --- | --- | --- | --- | --- | --- | --- | --- | --- |
|  |  | **Number tested** | **% NS** |  | **Number tested** | **% NS** |  | **Number tested** | **% NS** |
| Region 2 | NJ, NY | 29821 | 2.2% |  | 5359 | 13.4% |  | 404 | 53.5% |
| Region 3 | DE, DC, MD, PA, VA, WV | 8836 | 1.3% |  | 1306 | 10.0% |  | 98 | 16.3% |
| Region 4 | AL, FL, GA, KY, MS, NC, SC, TN | 72080 | 1.3% |  | 12752 | 15.5% |  | 1257 | 22.0% |
| Region 5 | IL, IN, MI, MN, OH, WI | 65437 | 1.2% |  | 10591 | 14.6% |  | 833 | 48.5% |
| Region 6 | AK, LA, NM, OK, TX | 43791 | 0.9% |  | 6123 | 17.1% |  | 462 | 37.7% |
| Region 9 | AZ, CA, HI, NV | 26909 | 1.0% |  | 4162 | 16.1% |  | 254 | 47.6% |
| Region 10 | AK, ID, OR, WA | 8422 | 0.4% |  | 1164 | 3.8% |  | 43 | 2.3% |
| Region 1,7,8^b^ | CT, ME, MA, NH, RI, VT, IA, KS, MO, NE, CO, MT, ND, SD, UT, WY | 10485 | 0.2% |  | 1423 | 8.6% |  | 63 | 9.5% |
| P value^c^ |  |  | <0.0001 |  |  | <0.0001 |  |  | <0.0001 |

Abbreviations: Carb, carbapenem; NS, non-susceptible

^a^US Territories were not included

^a^HHS Regions 1 (CT, ME, MA, NH, RI, VT), 7 (IA, KS, MO, NE), and 8 (CO, MT, ND, SD, UT, WY) were combined due to small group counts

^c^Overall testing for statistical difference in % Carb-NS among regions

**Supplementary Table S4.** Non-susceptibility of Isolates to Selected Antimicrobials.

| **Pathogen** | **Total Isolates** | | |  | **Carb-NS Isolates** | | | |  |
| --- | --- | --- | --- | --- | --- | --- | --- | --- | --- |
|  | **Number Tested** | **Number NS to Specified Anti-microbial** | **% NS** |  | | **Number of Carb NS Isolates** | **Number NS to Specified Anti-microbial** | **% NS** | |
| Enterobacteriaceae |  |  |  |  | |  |  |  | |
| ESC4 | 265,781 | 36,220 | 13.6 |  | | 3,227 | 2,493 | 77.3 | |
| Piperacillin-tazobactam | 265,781 | 20,772 | 7.8 |  | | 3,227 | 2,321 | 71.9 | |
| FQ3 | 265,781 | 71,968 | 27.1 |  | | 3227 | 1,851 | 57.4 | |
| MDR^a^ | 265,781 | 18,392 | 6.9 |  | | 3,227 | 2,504 | 77.6 | |
| *Pseudomonas aeruginosa* |  |  |  |  | |  |  |  | |
| ESC2 | 42,880 | 7,760 | 18.1 |  | | 6,256 | 3,427 | 54.8 | |
| Piperacillin-tazobactam | 42,880 | 4,490 | 10.5 |  | | 6,256 | 2,180 | 34.8 | |
| FQ2 | 42,880 | 12,044 | 28.1 |  | | 6,256 | 4,457 | 71.2 | |
| MDR^a^ | 42,880 | 5,564 | 13.0 |  | | 6,256 | 4,004 | 64.0 | |
| *Acinetobacter* spp. |  |  |  |  | |  |  |  | |
| ESC2 | 3,414 | 1,541 | 45.1 |  | | 1,215 | 1,047 | 86.2 | |
| Piperacillin-tazobactam | 3,414 | 956 | 28.0 |  | | 1,215 | 622 | 51.2 | |
| FQ2 | 3,414 | 1,660 | 48.6 |  | | 1,215 | 1,187 | 97.7 | |
| MDR^a^ | 3,414 | 1,483 | 43.4 |  | | 1,215 | 1,188 | 97.8 | |

Abbreviations: Carb, carbapenem; ESC, extended spectrum cephalopsorins; ESC2, cefepime, ceftazidime; ESC4, cefepime, cefotaxime, ceftazidime, ceftriaxone; FQ, fluoroquinolones; FQ2, levofloxacin and ciprofloxacin; FQ3, moxifloxacin, levofloxacin, and ciprofloxacin; MDR^a^, multidrug resistant (as defined by Weiner LM et al., Infect Control Hosp Epidemiol **2016**; 37:1288-1301); NS, non-susceptible

^a^MDR Enterobacteriaceae, intermediate susceptibility (I) or resistant (R) to at least 1 drug in 3 of the following 5 classes: ESC4, FQ3, aminoglycosides, carbapenems, and piperacillin or piperacillin/tazobactam

MDR *P. aeruginosa*, I or R to at least 1 drug in 3 of the following 5 classes: ESC2, FQ2, aminoglycosides, carbapenems (imipenem or meropenem), and piperacillin or piperacillin/tazobactam

MDR *Acinetobacter* spp., I or R to at least 1 drug in 3 of the following 6 classes: ESC2, FQ2, aminoglycosides, carbapenems (imipenem, meropenem, or ertapenem), piperacillin or piperacillin/tazobactam, and ampicillin/sulbactam

**Supplementary Figure 1.** Adjusted effect^a^ of ICU on Carb-NS overall and by pathogen. Horizontal bars indicate 95% confidence intervals.

Abbreviations: Carb, carbapenem; CI, confidence interval; ICU, intensive-care unit; NS, non-susceptible.

^a^Effects were adjusted for onset, source, pathogen, and hospital characteristics (teaching status, bed size, urban/rural, geographic region) using generalized linear mixed models
